# Strain differences in the extent of brain injury in mice after tetramethylenedisulfotetramine-induced *status epilepticus*

**DOI:** 10.1016/j.neuro.2021.08.011

**Published:** 2021-08-31

**Authors:** Jonas J. Calsbeek, Eduardo A. González, Casey A. Boosalis, Dorota Zolkowska, Donald A. Bruun, Douglas J. Rowland, Naomi H. Saito, Danielle J. Harvey, Abhijit J. Chaudhari, Michael A. Rogawski, Joel R. Garbow, Pamela J. Lein

**Affiliations:** a Department of Molecular Biosciences, University of California, Davis, School of Veterinary Medicine, Davis, CA, 95616, USA; b Department of Neurology, University of California, Davis, School of Medicine, Davis, CA, 95616, USA; c Center for Molecular and Genomic Imaging, University of California, Davis, College of Engineering, Davis, CA, 95616, USA; d Department of Public Health Sciences, University of California, Davis, School of Medicine, Davis, CA, 95616, USA; e Biomedical Magnetic Resonance Laboratory, Mallinckrodt Institute of Radiology, Washington University in St. Louis, School of Medicine, St. Louis, MO, 63110, USA

**Keywords:** MRI, Neurodegeneration, Neuroinflammation, PET, Tetramine

## Abstract

Acute intoxication with tetramethylenedisulfotetramine (TETS) can trigger *status epilepticus* (SE) in humans. Survivors often exhibit long-term neurological effects, including electrographic abnormalities and cognitive deficits, but the pathogenic mechanisms linking the acute toxic effects of TETS to chronic outcomes are not known. Here, we use advanced *in vivo* imaging techniques to longitudinally monitor the neuropathological consequences of TETS-induced SE in two different mouse strains. Adult male NIH Swiss and C57BL/6J mice were injected with riluzole (10 mg/kg, i.p.), followed 10 min later by an acute dose of TETS (0.2 mg/kg in NIH Swiss; 0.3 mg/kg, i.p. in C57BL/6J) or an equal volume of vehicle (10% DMSO in 0.9% sterile saline). Different TETS doses were administered to trigger comparable seizure behavior between strains. Seizure behavior began within minutes of TETS exposure and rapidly progressed to SE that was terminated after 40 min by administration of midazolam (1.8 mg/kg, i.m.). The brains of vehicle and TETS-exposed mice were imaged using *in vivo* magnetic resonance (MR) and translocator protein (TSPO) positron emission tomography (PET) at 1, 3, 7, and 14 days post-exposure to monitor brain injury and neuroinflammation, respectively. When the brain scans of TETS mice were compared to those of vehicle controls, subtle and transient neuropathology was observed in both mouse strains, but more extensive and persistent TETS-induced neuropathology was observed in C57BL/6J mice. In addition, one NIH Swiss TETS mouse that did not respond to the midazolam therapy, but remained in SE for more than 2 h, displayed robust neuropathology as determined by *in vivo* imaging and confirmed by FluoroJade C staining and IBA-1 immunohistochemistry as readouts of neurodegeneration and neuroinflammation, respectively. These findings demonstrate that the extent of injury observed in the mouse brain after TETS-induced SE varied according to strain, dose of TETS and/or the duration of SE. These observations suggest that TETS-intoxicated humans who do not respond to antiseizure medication are at increased risk for brain injury.

## 1. Introduction

Tetramethylenedisulfotetramine (TETS) is a globally banned rodenticide listed by the United States Department of Homeland Security as a potential chemical threat agent (Shakarjian et al., 2016). Worldwide production of TETS was banned in 1984 because of its extremely low LD_50_ of 100 μg/kg, which is approximately 7–10 mg in a 70 kg human, and because of its persistence in the environment (Guan et al., 1993, reviewed in Whitlow et al., 2005). Nevertheless, because of high consumer demand for effective rodenticides, TETS, which is easy and inexpensive to synthesize, remains widely available on the black market in Asian countries (reviewed in Patocka et al., 2018). Accidental and intentional poisonings are not uncommon in Asian countries, and typically occur through ingestion since TETS is tasteless and odorless (reviewed in Zhang et al., 2011). Mild symptoms including nausea, dizziness, and headache may occur with low dose exposures while high dose exposures are associated with pulmonary edema, organ congestion, calcium depositions in the kidney, fatty liver, and seizures that can progress to *status epilepticus* (SE) (Zhou et al., 1998). Acute intoxication with high doses can be fatal within a few hours (Barrueto et al., 2003). Brainstem hemorrhage, cerebral edema, recurrent seizures and cognitive deficits have been observed in patients who survived acute poisoning with TETS (reviewed in Whitlow et al., 2005).

The mechanism by which TETS triggers seizures involves GABA_A_ receptor (GABA_A_R) antagonism (Pressly et al., 2021, 2018; Shakarjian et al., 2016). The current standard of care for terminating TETS-induced seizure activity is treatment with GABA_A_R positive allosteric modulators, typically, high dose benzodiazepines (Whitlow et al., 2005). While this treatment is sufficient to prevent death, it does not protect against the long-term electrographic abnormalities, including recurrent seizures and cognitive deficits (Croddy, 2004; Deng et al., 2012; Whitlow et al., 2005). It remains unclear whether these neurological deficits are the result of excitotoxicity downstream of prolonged seizure activity or the consequence of seizure-independent toxicodynamic actions of TETS. This uncertainty reflects the paucity of clinical and preclinical data regarding the pathogenic mechanisms that underlie the long-term consequences of TETS-induced SE. To address this data gap, rodent models of TETS-induced seizures have been developed to study the consequences of acute TETS exposure (Rice et al., 2017; Shakarjian et al., 2012; Zolkowska et al., 2012). However, in these early models of TETS-induced seizures, animals did not develop SE. For example, in the NIH Swiss mouse, during the first 18–20 min following TETS exposure, mice experienced 2–3 brief clonic seizures followed by a tonic-hind limb extension and death (Pessah et al., 2016; Zolkowska et al., 2012). Administration of high dose benzodiazepine or low dose benzodiazepine and neurosteroid with GABA_A_ receptor modulatory activity prior to tonic-hind limb extension rescued animals from death (Flannery et al., 2015; Vito et al., 2014). In these TETS-induced seizure models, neurodegeneration was not detected by FluoroJade B staining at 1, 2, 3, or 7 days post-exposure, but there was a transient neuroinflammatory response, evident as increased IBA-1 and GFAP immunoreactivity in the cortex and hippocampus during the first 1–3 days post-exposure (Zolkowska et al., 2012). These animals also did not exhibit changes in cognitive, anxiety-like, or depression-like behaviors (Flannery et al., 2015). The failure of these models to recapitulate the chronic effects observed in humans who survive acute TETS intoxication was attributed to the fact that mice did not develop SE using these dosing paradigms.

We recently developed a mouse model of TETS-induced SE in which animals are injected with riluzole 10 min prior to TETS to protect against the rapidly lethal effects of the toxicant (Pessah et al., 2016; Zolkowska et al., 2018). Riluzole is a benzothiazole that blocks glutamatergic signaling in the nervous system *via* inactivation of voltage-gated sodium ion channels (Doble, 1996). We previously demonstrated that pretreatment with riluzole blocks the hindlimb extension induced by TETS, which we believe is how riluzole promotes prolonged seizure activity following TETS intoxication while protecting all mice from acute lethality (Zolkowska et al., 2018). In this model, TETS-intoxicated animals experienced persistent seizures that began within minutes of TETS injection and continued until the animal died, usually within 60–90 min post-exposure. Treatment with a benzodiazepine at 40–60 min post-exposure rescued most (≥95%) animals exposed to TETS using this modified dosing paradigm. Here, we leveraged this novel mouse model to determine whether TETS-induced SE is associated with robust and persistent neurodegeneration and neuroinflammation as has been documented in preclinical models of organophosphate-induced SE (reviewed by Andrew and Lein, 2021; Naughton and Terry, 2018; Tsai and Lein, 2021). Additionally, while preclinical models of TETS-induced seizures have primarily used NIH Swiss or C57BL/6 mice, these two strains have not been directly compared, an important consideration given the well-known influence of strain on mouse susceptibility to seizurogenic triggers (Copping et al., 2019). The overall goal of this study was to use *in vivo* imaging techniques to quantify the spatiotemporal patterns of neuropathology after TETS-induced SE and to determine whether these patterns varied between two mouse strains commonly used to study seizure disorders. We have previously validated magnetic resonance imaging (MRI) and positron emission tomography (PET) for monitoring spatiotemporal profiles of brain damage in preclinical models of chemical-induced SE (Hobson et al., 2019, 2017). Here, we used longitudinal three-dimensional, T_2_-weighted (T2W) anatomic MR images of the brain to track the spatiotemporal changes in brain or ventricle volume, and diffusion-weighted imaging (DWI) to document changes in regional tissue water diffusion that distinguish healthy *versus* pathologic tissue (Hobson et al., 2017). For PET we employed a radiolabeled ligand of the translocator protein (TSPO), which has been shown to be a biomarker of neuroinflammation in clinical and preclinical studies (Guilarte, 2019).

## 2. Materials and methods

### 2.1. Animals

All studies involving animals were conducted in accordance with protocols approved by the University of California, Davis, Institutional Animal Care and Use Committee to minimize pain and suffering. All animals were housed in facilities accredited by AAALAC International. Adult male C57BL/6J mice (8–10 weeks old; 22–33 g) and NIH Swiss mice (8–10 weeks old; 22–33 g) were maintained on a 12:12 light:dark cycle in a temperature and humidity-controlled vivarium (22 ± 2 °C; 40–50% humidity). Mice were singly housed in standard plastic cages, provided standard mouse chow (LabDiet, #5058) and tap water *ad libitum*, and allowed to acclimate for at least 7 days prior to experimentation.

### 2.2. Drugs and dosing paradigm

**Fig. 1.**
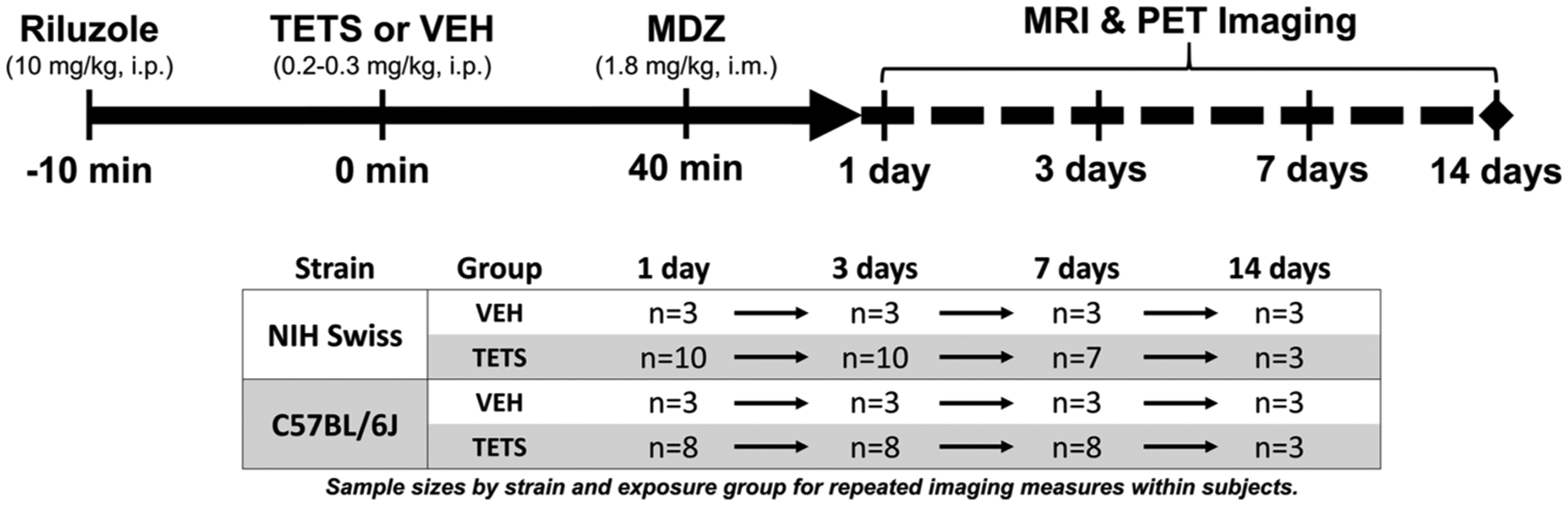
Dosing paradigm and imaging schedule. Adult mice were pretreated with riluzole 10 min prior to the administration of vehicle or tetramethylenedisulfotetramine (TETS). NIH Swiss mice were injected with TETS (0.2 mg/kg) or VEH, and C57BL/6J were injected with TETS (0.3 mg/kg) or VEH. 40 min later, a rescue dose of midazolam (MDZ) was administered to stop seizure behavior. Brains of TETS-intoxicated and vehicle control mice were imaged using MRI and PET at 1, 3, 7, and 14 days post exposure. Sample sizes for each strain and exposure group are shown in the table to indicate repeated imaging time points within subjects for mice in this study.

Riluzole was purchased from Oakwood Products (West Columbia, SC, USA) and recrystallized to increase purity (>98%). TETS was synthesized and its purity (>99%) determined as previously described (Zhao et al., 2014). Pharmaceutical grade midazolam (MDZ; 5 mg/mL MDZ in 0.8% sodium chloride, 0.01% edetate disodium, 1% benzyl alcohol) was purchased from Hospira (Lake Forest, IL, USA). Mice were injected with riluzole (10 mg/kg, i.p.; Fig. 1) 10 min prior to injection with TETS (0.2–0.3 mg/kg, i.p.), a dosing paradigm previously shown to induce SE in mice (Pessah et al., 2016; Zolkowska et al., 2018). Pilot studies were conducted to identify a dose of TETS that caused SE with similar onset and severity in NIH Swiss (0.2 mg/kg, i.p.) and C57BL/6J (0.3 mg/kg, i.p.). Animals were administered MDZ (1.8 mg/kg, i.m.) 40 min post-TETS to terminate seizure behavior. Vehicle animals were similarly treated with riluzole and MDZ, but injected with vehicle (10% DMSO in 0.9% sterile saline) in place of TETS. Animals looking weak or ill following dosing were placed on heating pads and injected with 1 mL dextrose (10%, s.c.) as needed.

### 2.3. In vivo imaging and analysis

#### 2.3.1. Magnetic resonance imaging (MRI)

MRI scans were performed at the Center for Molecular and Genomic Imaging (CMGI) at the University of California, Davis, using a Bruker Biospec 70/30 (7 T) preclinical MR scanner (Bruker BioSpin MRI; Ettlingen, Germany). Whole brain anatomical T2W and diffusion-weighted MRI data were collected from mice at 1, 3, 7, and 14 days after TETS exposure, similar to what has been previously described (Hobson et al., 2017). MRI was performed using a 116 mm internal diameter B-GA12S gradient coil (450 m T/m, 4500 T/m/s), a 72-mm internal diameter linear transmit RF coil, and a four-channel mouse brain surface coil for signal reception. Mice were continuously anesthetized with isoflurane (1–3%) in medical air, which was adjusted to maintain a respiration rate of 70–90 breaths per min and were secured in a stereotactic restraint to prevent motion. Warm air was used to maintain body temperature (37 °C) during image acquisition. T2W rapid acquisition with repeated echoes (RARE) images were acquired for ~12 min using the following parameters: repetition time (TR) = 6100 ms; effective echo time (TE) = 60 ms; RARE factor = 8; averages = 8; field of view (FOV) = 15 × 15 mm^2^ (120 × 120 data matrix); 44 slices with 0.25 mm thickness. Diffusion scans were acquired with echo planar imaging (EPI) utilizing 8 segments and a diffusion strength of b = 1600s/mm^2^ to obtain apparent diffusion coefficient (ADC) scans using three diffusion directions. ADC scans immediately followed the T2W acquisition using the following parameters: TR = 5500 ms; TE = 30 ms; FOV = 17.5 × 15 mm^2^ (140 × 120 data matrix); 13 slices with 0.5 mm thickness; averages = 4. Paravision 5.1 software (Bruker BioSpin MRI) was used for image acquisition and reconstruction, and ADC maps were generated from diffusion images using a custom-built ImageJ macro.

#### 2.3.2. Positron emission topography (PET)

PET imaging was performed using a radiolabeled ligand ([^18^F] PBR111) that targets TSPO 18 kDa, a validated marker of neuroinflammation (Ottoy et al., 2018; Van Camp et al., 2010). Automated synthesis of [^18^F]PBR111 was performed as previously described (Bourdier et al., 2012). PET scans were performed at the CMGI at the University of California, Davis, using a Siemens Inveon DPET small animal scanner (Siemens Corporation; Munich, Germany) or a microPET Focus 120 (Siemens Corporation), as previously described (Hobson et al., 2019). For repeated measures in the same animal at different times post-exposure, animals were scanned on the same PET scanner. Mice were continuously anesthetized with isoflurane (1–3%) mixed with medical air, which was adjusted to maintain a respiration rate of 70–90 breaths per min during acquisition. Mice were secured in a stereotactic restraint to minimize motion during PET scans, and whole brain scans were acquired at 1, 3, 7, and 14 days post-exposure. Immediately following the beginning of data acquisition, a bolus of [^18^F]PBR111 (avg activity = 39.6 ± 2.2 MBq; in 200 μL saline) was injected *via* tail-vein, and PET scan acquisition duration was 60 min.

#### 2.3.3. Image analysis

Image processing and analysis were performed using PMOD v.3.9 (PMOD Technologies; Zurich, Switzerland). A mouse brain digital atlas (Mirrione et al., 2007) was used to isolate individual brain regions in three dimensions for each subject and was manually adjusted for regional accuracy and registered to the T2W and diffusion MR scans. ADC values were extracted, initially calculated on a voxel-by-voxel basis, and averaged for each brain region of interest. The standard deviation of the ADC (‘ADC SD’) between voxels in each brain region was also calculated and provided a useful metric for quantifying neurodegeneration (Hobson et al., 2017). PET scans were registered to the T2W reference image to extract the standardized uptake value (SUV) for 30–60 min of data for each brain region. The SUV averaged over each brain region (‘Average SUV’) served as a measure of neuroinflammation.

### 2.4. Histology

At 7 days post-TETS exposure, a subset of mice imaged in this study was anesthetized with 4–5% isoflurane in medical-grade oxygen, perfused with ~25 mL of 4% (w/v) paraformaldehyde (PFA; Sigma; St. Louis, MO, USA) in phosphate-buffered saline (PBS; 3.6 mM Na_2_HPO_4_, 1.4 mM NaH_2_PO_4_, 150 mM NaCl; pH 7.2) using a Peri-Star Pro peristaltic pump (5 mL/min). Brains were removed, and the freshly perfused whole brains were immediately placed into 20 mL glass scintillation vials containing 10 mL of 4% (w/v) PFA in PBS (pH 7.2). After 24 h, whole brains were transferred into 30% (w/v) sucrose (Fisher; Houston, TX, USA) in PBS (3.6 mM Na_2_HPO_4_, 1.4 mM NaH_2_PO_4_, 150 mM NaCl; pH 7.2) for an additional 48 h or until the tissue sank completely to the bottom of the vial. Fixed whole brains were serially sectioned into 2-mm thick coronal slices using a mouse brain matrix (Zivic Instruments, #5325; Pittsburgh, PA, USA) and embedded by flash freezing in optimal cutting temperature medium (OCT; Fisher HealthCare; Waltham, MA, USA). Blocked brain sections were stored at −80 °C until cryosectioned using a Microm HM550 cryostat (Thermo Fisher Scientific, Waltham, MA, USA) into 10-μm thick slices on Superfrost plus microscope slides (Fisher HealthCare). Slides were stored at −80 °C prior to staining or immunohistochemistry.

#### 2.4.1. FluoroJade C (FJC) staining

Neurodegeneration was assessed using FJC (AG325, MilliporeSigma; Burlington, MA, USA) staining. Brain slices were thawed on ice, then incubated in 0.06% (w/v) KMnO_4_ (Sigma) in distilled H_2_O (dH_2_O) for 10 min, rinsed 3x in dH_2_O, and incubated in 0.00015%, w/v FJC and 0.5 μg/mL DAPI (Invitrogen; Carlsbad, CA, USA) in 0.1% acetic acid (Acros Organics; Geel, Belgium) in dH_2_0 for 10 min in the dark. Slides were then dipped in xylene (X5SK-4, Assay grade; Thermo Fisher Scientific) for 1 min, mounted in Permount (Thermo Fisher Scientific), coverslipped, and imaged at 20X magnification on a high content ImageXpress imaging system (Molecular Devices; Sunnyvale, CA, USA). Neurodegeneration of the cortex and hippocampus was evaluated by counting neurons positively labeled with FJC (per mm^2^) at 7 days post-exposure, as described previously (Zolkowska et al., 2012).

#### 2.4.2. Immunohistochemical analyses of neuroinflammation

Neuroinflammation was assessed by quantifying ionized calcium binding adaptor molecule 1 (IBA-1) immunoreactivity, a biomarker of microglia (Ito et al., 1998), as previously described (Guignet et al., 2020). Briefly, brain slices were incubated in blocking buffer (PBS containing 10% normal goat serum (v/v; Vector Laboratories, Burlingame, CA, USA), 1% bovine serum albumin (w/v; Sigma), and 0.03% Triton (v/v; ThermoFisher Scientific) for 1 h at room temperature to prevent nonspecific binding of the primary antibody. Brain slices were then incubated in primary rabbit anti-IBA-1 antibody (1:1000 in blocking buffer; RRID:AB_839504, Wako Laboratory Chemicals; Richmond, VA, USA) overnight at 4 °C. At the end of incubation, brain slices were washed 3x for 5 min with PBS and then incubated in secondary antibody solution (Goat anti-rabbit IgG AlexaFluor 568 diluted 1:500 in PBS containing 0.03% Triton (v/v); RRID:AB_10563566, Life Technologies; Carlsbad, CA, USA) for 1 h at room temperature in the dark. Slides were then mounted in ProlongGold anti-fade reagent with DAPI (Thermo Fisher Scientific), coverslipped, and imaged on a high content ImageXpress imaging system at 20X magnification. Neuroinflammation in the cortex and hippocampus of all animals was evaluated by measuring the percent area of brain tissue positively labeled with IBA-1 at 7 days post-exposure, as described previously (Zolkowska et al., 2012).

### 2.5. Statistical analyses

Primary outcomes from the *in vivo* imaging studies included regional measures of the standard deviation (SD) of the ADC value and average SUV. Regions of interest (ROIs) included the amygdala, hippocampus, piriform cortex, somatosensory cortex, striatum, and thalamus. The ADC, ADC SD, and average SUV were available for all ROIs in all animals. Measures from the right and left hemisphere were averaged for the amygdala, hippocampus, piriform cortex, and striatum. Data were available for two strains of mice (C57BL/6J, NIH Swiss), for two treatment groups (TETS, vehicle), and at various days post-exposure (1, 3, 7, 14). Days post-exposure was treated as a categorical variable to allow for comparisons between experimental groups by day. Due to the repeated measures across brain regions and days post-exposure, mixed effects regression models were used to assess differences between TETS and vehicle. Both the average SUV and the ADC SD were transformed using the natural log prior to analysis to meet assumptions of the model, including normality and constant variance. All models considered day post-exposure, brain region, treatment group and their interactions, and included an animal-specific random effect. Akaike Information Criterion was used for model selection. Contrasts specifically comparing experimental groups overall, by treatment or by day, were constructed and used to test for differences between TETS and vehicle. Benjamini-Hochberg False Discovery Rate (FDR) was used to control for multiple comparisons across all contrasts within a model (Benjamini and Hochberg, 1995). Geometric mean ratios (GMR) and corresponding 95% confidence intervals (CI) were used to quantify differences between exposure groups; if the confidence interval included 1, no statistically significant difference was observed between the groups. Primary analyses were conducted separately for each mouse strain, although secondary analyses directly assessed differences between strains, by including strain and related interactions in the models. For secondary analyses, 90% CI were obtained for the ratio of the GMR between strains to assess equivalence of the strains. All analyses were performed using SAS software (version 9.4, SAS Institute, Inc.; Cary, NC, USA), and graphics were created in R (version 3.6.3, R Core Team, Vienna, Austria).

## 3. Results

### 3.1. Diffusion-weighted MRI

**Fig. 2.**
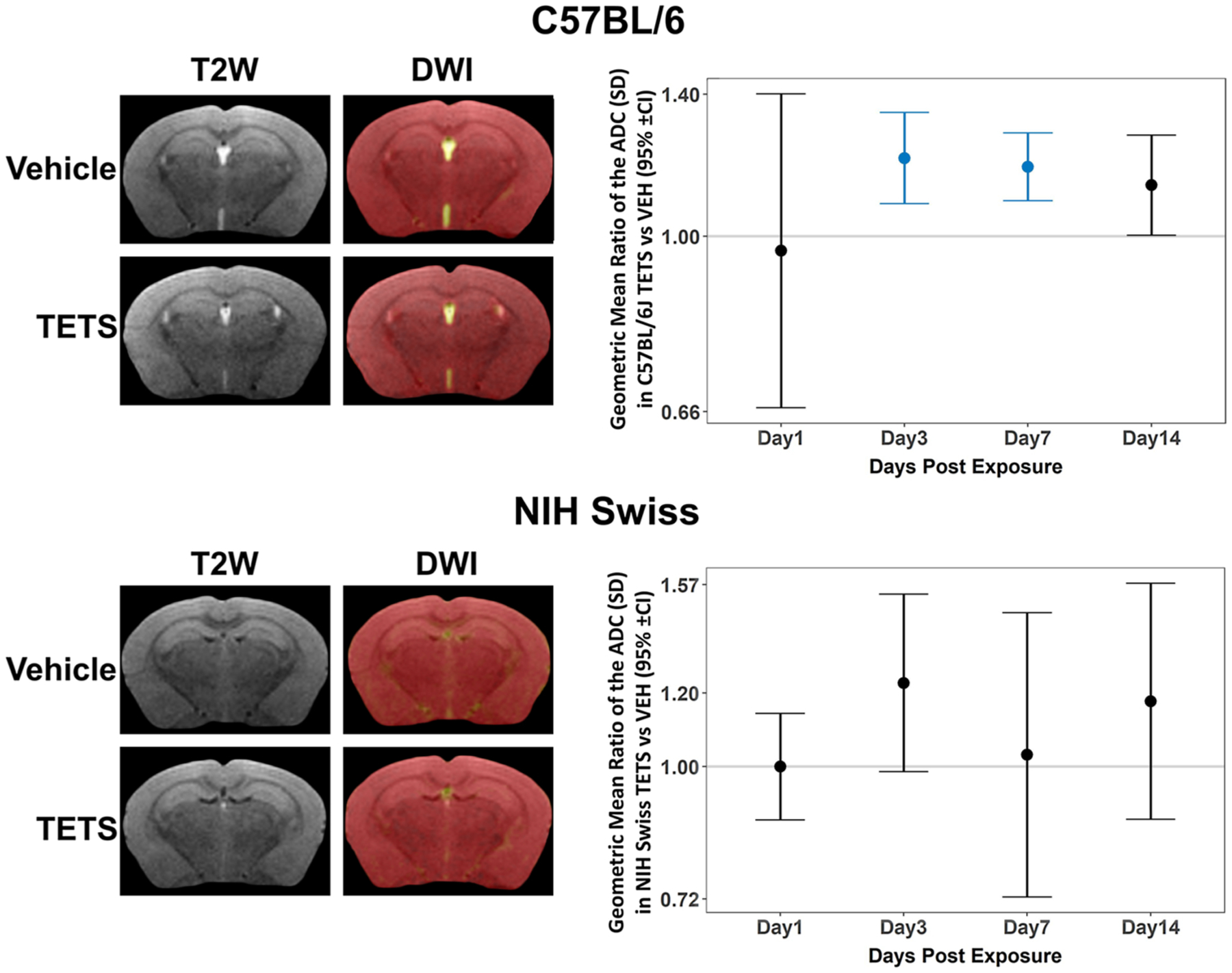
Acute TETS intoxication-induced neuropathology, as detected by diffusion-weighted imaging (DWI). Representative anatomical (T2W, left column) and parametric maps (DWI, right column) of vehicle (VEH) and TETS-intoxicated C57BL/6J and NIH Swiss mice. Geometric mean ratio (dot) of the standard deviation (SD) of the apparent diffusion coefficient (ADC SD) average for TETS *vs*. VEH mice with 95% confidence intervals (bars). Confidence intervals that do not include 1 (gray line) indicate a significant difference between TETS and VEH (those identified in turquoise survived the FDR correction). All VEH groups had n = 3 for each time point. Sample sizes for TETS time points: Day 1: n = 8–9; Day 3: n = 8–9; Day 7: n = 7–8; Day 14: n = 3. Individual data points used to generate this figure can be found in the supplemental material (Tables S1 and S2).

For both strains of mice, exposure effects were not dependent on brain region, so estimates were collapsed across regions in the statistical analyses (Fig. 2). For the C57BL/6J mice, the standard deviation (SD) of the ADC value was significantly higher in TETS animals than vehicle (VEH) animals on days 3 (p < 0.001), 7 (p < 0.001), and 14 (p < 0.05) post-exposure, but the difference on day 14 did not survive the FDR correction. There was no difference between groups on day 1 (p = 0.8). For the NIH Swiss mice, after FDR correction, there were no significant differences in the ADC SD between TETS and VEH animals on any day. Individual data points used to generate this figure are available in the supplemental material (Tables S1 and S2).

### 3.2. TSPO PET imaging

**Fig. 3.**
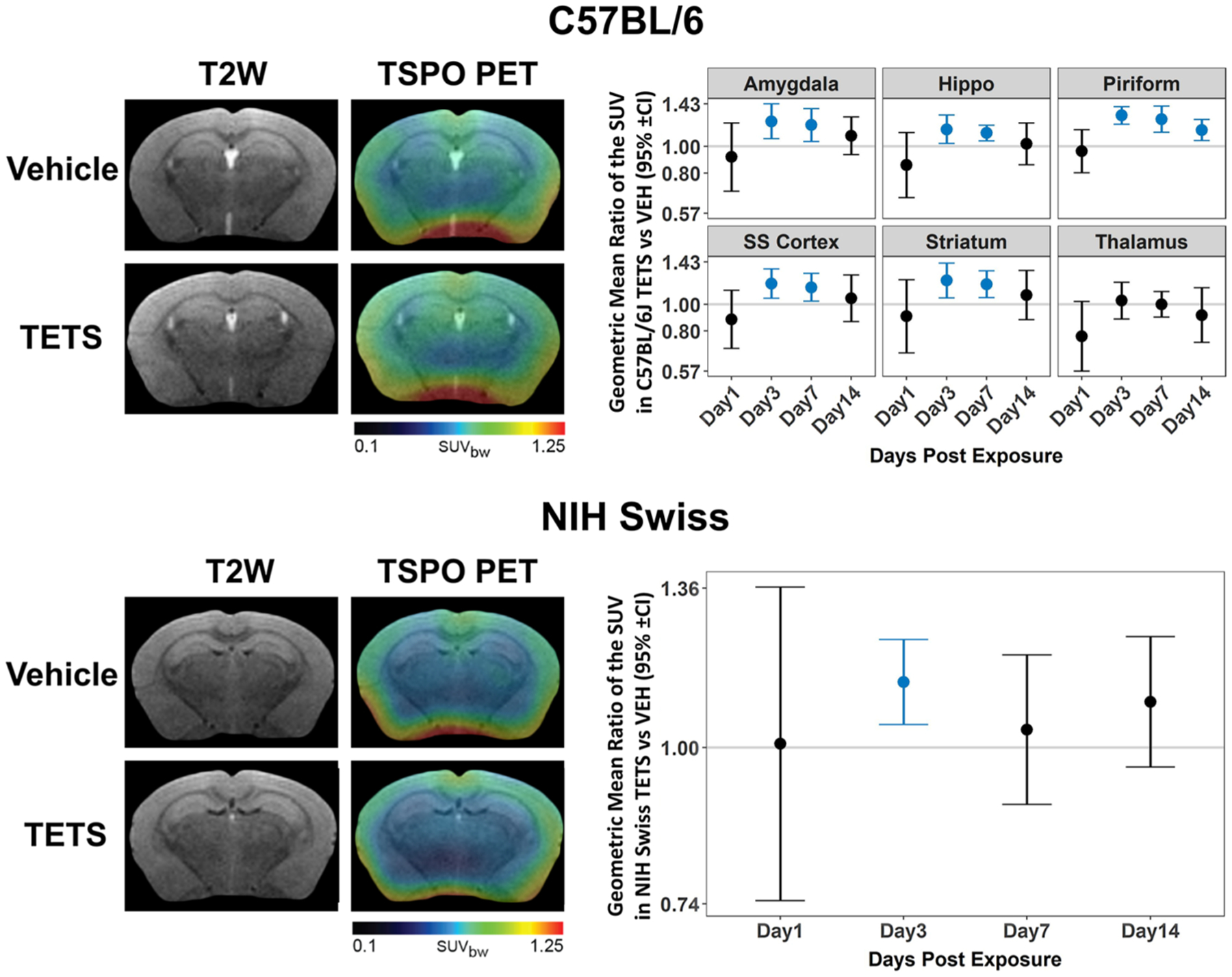
Acute TETS intoxication-induced neuroinflammation, as detected by TSPO PET imaging. Representative anatomical (T2W, left column) and parametric maps (TSPO PET, right column) of vehicle (VEH) and TETS-intoxicated C57BL/6J and NIH Swiss mice. Geometric mean ratio (dot) of the TSPO standard uptake value (SUV) for TETS *vs*. VEH mice with 95% confidence intervals (bars). No statistically significant differences were detected between brain regions for NIH Swiss mice, so these data were collapsed. Confidence intervals that do not include 1 (gray line) indicate a significant difference between TETS and VEH (those identified in turquoise survived the FDR correction). All VEH groups had n = 3 for each time point. Sample sizes for TETS time points: Day 1: n = 8–9; Day 3: n = 8–9; Day 7: n = 7–8; Day 14: n = 3. Individual data points used to generate this figure can be found in the supplemental material (Table S3).

There were no significant differences in the average SUV between C57BL/6J TETS and VEH mice in any brain region on day 1 post exposure (Fig. 3). However, on days 3 and 7 post-exposure, the average SUV was significantly higher in TETS than VEH animals across all brain regions (p < .02) except the thalamus. On day 14 post-exposure, average SUV remained significantly higher in TETS than VEH animals in the piriform cortex only (Geometric mean ratio (GMR) = 1.1, 95% CI: (1.05, 1.25), p = .003). In NIH Swiss mice, estimates of differences were collapsed across brain regions because exposure effects were not region dependent. Average SUV was significantly higher in TETS animals than VEH animals on day 3 only (GMR: 1.1, 95% CI: (1.04, 1.23), p = .003). Individual data points used to generate this figure can be found in the supplemental material (Table S3).

### 3.3. Strain comparison

TETS exposure effects were not significantly different between mouse strains for any outcome. In C57BL/6J mice, the exposure effect measured by SUV was 13% lower than that in NIH Swiss mice on day 1 (ratio of GMR = 0.87; 90% CI: 0.63, 1.21; p = .5), 4–5% higher on day 3 (ratio of GMR = 1.05; 90% CI: 0.94, 1.16; p = .5), 11% higher on day 7 (ratio of GMR = 1.11; 90% CI: 0.97, 1.27; p = .2) and 4.0% lower on day 14 (ratio of GMR = 0.96; 90% CI: 0.81, 1.14; p = .7). Similarly, the effect measured by ADC SD was 1.0% higher in C57BL/6J mice than NIH Swiss mice (ratio of GMR = 1.01; 90% CI: 0.88, 1.15; p = .9).

### 3.4. Seizure duration may influence neuropathology

**Fig. 4.**
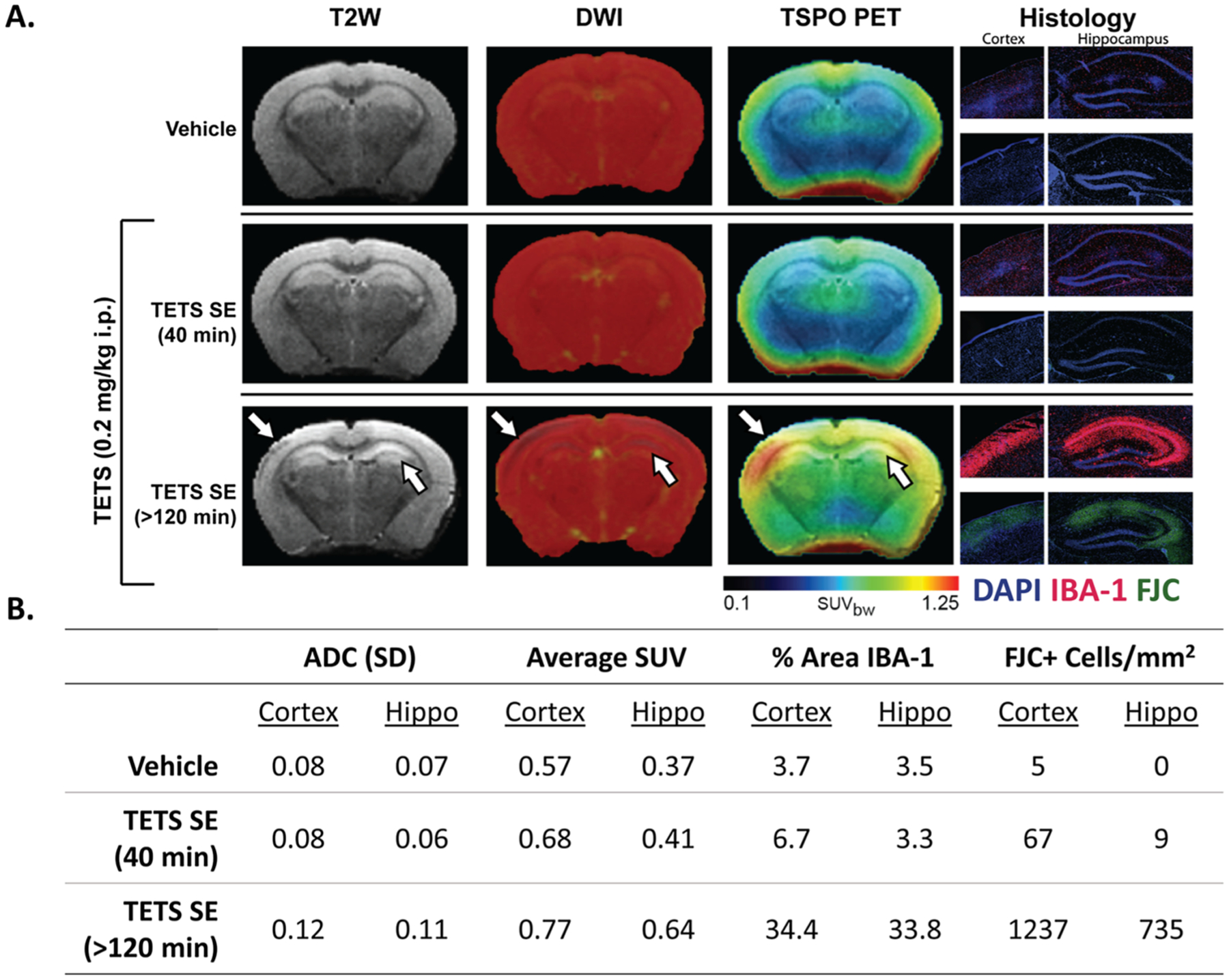
Seizure duration correlates with severity of neuropathology at 7 days post-exposure in NIH Swiss mice acutely intoxicated with TETS. (A) Representative anatomical T2W MRI (first column), diffusion-weighted MRI (second column), TSPO PET (third column), and histology (last columns) images from VEH mouse (top row), TETS-intoxicated mouse that experienced SE for 40 min (middle row), and TETS-intoxicated mouse that exhibited SE > 120 min (bottom row). The middle row shows a TETS animal that responded normally to rescue by midazolam at 40 min after seizure initiation (40 min seizure), whereas the bottom row is of a TETS animal that continued to seize after midazolam treatment yet survived (>120 min seizure). Arrows point to regions of hypointensity (T2W & DWI) and high radiotracer uptake (TSPO PET), indicating neurodegeneration and neuroinflammation, respectively. IBA-1 (red) identifies microglia; FJC (green), degenerating neurons; DAPI (blue), nuclei. (B) Quantitative data table reporting regional ADC SD, average PET SUV, % area IBA1-positive, and FJC-positive cells/mm^2^ from the vehicle (n = 1), TETS SE (40 min; n = 1), and prolonged TETS SE (>120 min; n = 1) mice.

During the course of this study, we observed one NIH Swiss mouse acutely intoxicated with TETS that continued to seize after MDZ treatment, and thus experienced SE > 120 min. This mouse survived for 7 days post-exposure. Histological analysis of the brain from this unique animal revealed abundant FJC labeling in the outer cortex and in the CA1 and CA3 regions of the hippocampus, demonstrating severe neurodegeneration. In contrast, no FJC-labeled cells were observed in the cortex or hippocampus of NIH Swiss mice 7 days after exposure to VEH or TETS with SE terminated at 40 min by midazolam (Fig. 4A). Quantitative analysis confirmed this observation with VEH, TETS SE 40 min, and TETS SE > 120 animals showing 5, 67, and 1237 FJC-positive cells per square millimeter in the cortex and 0, 9, and 735 FJC-positive cells in the hippocampus, respectively (Fig. 4B). The TETS animal who experienced SE > 120 min also had significantly increased IBA-1 immunoreactivity in the outer cortex and in the CA1 and CA3 regions of the hippocampus compared to VEH and TETS animals with SE for 40 min, suggesting an elevated neuroinflammatory response in this animal (Fig. 4A). This finding was confirmed by quantitative analysis of the percent area of regional IBA-1 staining: VEH, TETS SE 40 min, and TETS SE > 120 min animals showed areas of 3.7, 6.7, and 34.4% IBA-1 immunoreactivity in the cortex and 3.5, 3.3, and 33.8% IBA-1 immunoreactivity in the hippocampus, respectively (Fig. 4B).

T2W MRI revealed regions of hyperintensity in the cortex and hippocampus of the TETS animal with SE > 120 min compared to VEH and TETS animals with SE of 40 min, suggesting tissue degeneration with increased duration of SE. A parametric ADC map showed visibly lower levels of intensity in the outer cortex and hippocampus of the TETS SE > 120 min animal as well (Fig. 4A). ADC SD values in the VEH, TETS SE 40 min, and TETS SE > 120 min animals were 0.08, 0.08, and 0.12 in the cortex and 0.07, 0.06, and 0.11 in the hippocampus, respectively (Fig. 4B). The TSPO PET images show noticeably higher levels of radiotracer uptake in the cortex and hippocampus regions of the TETS animal with SE > 120 min compared to the VEH and TETS animal with SE of 40 min (Fig. 4A). Average SUV values of the VEH, TETS SE 40 min, and TETS SE > 120 min animals were 0.57, 0.68, and 0.77 in the cortex and 0.37, 0.41, and 0.64 in the hippocampus, respectively (Fig. 4B).

## 4. Discussion

The goal of this study was to use *in vivo* imaging to longitudinally monitor neuropathology in a novel mouse model of TETS-induced SE (Pessah et al., 2016; Zolkowska et al., 2018), and to compare quantitative outcomes between two mouse strains, NIH Swiss and C57BL/6J, that are commonly used as seizure models. Our major findings were: (1) Mice that experienced ~40 min of TETS-induced SE exhibited delayed and transient neuropathology; and (2) while statistically significant differences between strains were not observed, TETS-induced brain injury, as indicated by significantly increased ADC SD was observed only in C57BL/6J mice, and neuroinflammation, measured as increased SUV in TSPO PET, was observed at 3 and 7 days post-exposure in the C57BL/6J mice but only at 3 days post-exposure in the NIH Swiss mouse. One interesting observation from this study was of an unusual TETS-intoxicated NIH Swiss mouse that survived SE for at least 120 min. Both *in vivo* imaging and histologic assessments documented significant neurodegeneration and a more robust neuroinflammatory response at 7 days post-exposure in this mouse compared to NIH Swiss mice with TETS-induced SE of ~40 min. While conclusions cannot be drawn from this single animal, it suggests that the duration of TETS-induced SE may increase the extent of neuropathology.

Previous studies demonstrated that acute intoxication with TETS can trigger dose-dependent seizures and death in NIH Swiss (Zolkowska et al., 2012) and C57BL/6 (Shakarjian et al., 2015, 2012) mice, but these exposures did not cause SE. NIH Swiss mice that experienced only brief clonic seizures after exposure to TETS (0.15 mg/kg, i.p.) presented with significantly increased expression of GFAP and IBA-1 at 1–3 days post-exposure in cortex and hippocampus, but no neurodegeneration or behavioral deficits (Flannery et al., 2015; Vito et al., 2014). The absence of severe neuropathology associated with these TETS exposures was attributed to the fact that these animals did not develop SE. Recently, we found that pretreatment with riluzole to prevent tonic hindlimb extension caused mice acutely intoxicated with TETS to transition from clonic-tonic seizures to prolonged SE that developed within minutes after exposures and persisted until animals died, typically 60–90 min post-exposure (Pessah et al., 2016; Zolkowska et al., 2018). In this model of TETS-induced SE, mice were rescued from death by administration of various anti-seizure treatments at 40 min following TETS injection (Pessah et al., 2016; Zolkowska et al., 2018).

Preclinical studies have demonstrated that 20–40 min of SE triggered by organophosphates (OPs) caused significant neuroinflammation and neurodegeneration in multiple brain regions that persisted for days to months [reviewed by (de Araujo Furtado et al., 2012; Guignet and Lein, 2019)]. Thus, we hypothesized that animals surviving ~40 min of SE after acute TETS intoxication would show extensive brain injury detectable by MRI and TSPO PET as previously reported for a rat model of OP-induced SE (Hobson et al., 2019, 2017). In our earlier histologic evaluation of neurodegeneration in TETS-intoxicated NIH Swiss mice that did not develop SE, no neurodegeneration was observed by FluoroJade B staining in any brain region from 4 h to 7 days post-exposure (Zolkowska et al., 2012). The NIH Swiss mouse model of TETS SE appeared to have greater brain damage than observed in the earlier model as evidenced by a significantly increased ADC SD from diffusion-weighted MRI at 3 days post-exposure. While a direct comparison between these two models is not possible because different methods were used to assess brain damage, our previous studies in a rat model of OP-induced SE has demonstrated a strong positive correlation between the ADC SD and FJC labeling (Hobson et al., 2017). Similar to observations from the earlier TETS seizure model, the novel TETS SE NIH Swiss model exhibited increased neuroinflammation at 3 days post-exposure, although again, different methods (GFAP and IBA-1 immunohistochemistry *vs*. TSPO PET) were used to detect neuroinflammation. Notably, the magnitude and persistence of brain injury detected by diffusion-weighted MRI and TSPO PET in the TETS SE mouse models was less severe compared to that reported in OP SE rat models (Hobson et al., 2019, 2017). The reasons for these differences in neuropathology between TETS-induced and OP-induced SE models are not known. Possibilities include differences in species, the nature of electrographic changes during SE (Calsbeek et al., 2018), seizure-independent toxicodynamics (*e.g.*, acetylcholinesterase inhibition by OPs *vs.* GABA_A_ receptor inhibition by TETS), or more effective termination by midazolam of SE triggered by TETS (Zolkowska, unpublished data) *vs*. OPs (Supasai et al., 2020; Wu et al., 2018). Collectively, however, the observations from this study suggest that, in contrast to OP-induced SE, TETS-induced SE is not necessarily associated with extensive brain damage.

Consistent with observations from preclinical models of OP-induced SE (Baille et al., 2005; McDonough et al., 1995; Siso et al., 2017), our observations suggest that the duration of TETS-induced SE influences the extent of neuropathology. While administration of midazolam at 40 min post-TETS injection terminated persistent seizures in almost all (≥95%) mice in our study, one NIH Swiss mouse continued to seize for ≥80 min after treatment with midazolam for a total SE duration ≥120 min. Analyses of brain damage at 7 days post-exposure by *in vivo* imaging and histology (FJC staining to detect neurodegeneration and IBA-1 immunohistochemistry to detect neuroinflammation) revealed greater neuropathology in this mouse than was observed in midazolam-responsive NIH Swiss mice that experienced SE for ~40 min. A major limitation of this observation is that we only had one animal with SE ≥120 min, therefore, no statistically significant conclusions can be drawn. However, the magnitude of the differences in the severity of neuropathology between the animals with 40 min *vs.* 120 min of SE support a correlation between seizure duration and neuropathology following TETS-induced SE.

In mice, seizure response and behavioral outcomes following seizures are influenced by genetic background (Copping et al., 2019). Therefore, another key question addressed in this study was whether the neuropathologic consequences of TETS-induced SE differed between NIH Swiss and C57BL/6J mice, the two strains previously used to investigate TETS-induced seizures (Shakarjian et al., 2015, 2012; Zolkowska et al., 2012). Despite the lack of statistically significant differences in direct comparison of *in vivo* imaging metrics between strains, the presence and time course of brain damage measured by MRI and PET varied by strain. Exposure effects between VEH and TETS NIH Swiss mice were significantly different only for PET and only on day 3 post-exposure, whereas TETS-intoxicated C57BL/6J were found to be significantly different from VEH as determined by MRI and PET at days 3 and 7 post-exposure, suggesting that genetic background may influence the neuropathologic consequences of TETS-induced SE. It is noteworthy that the dose of TETS used in the C57BL/6J mice (0.3 mg/kg TETS) was modestly greater than that used in the NIH Swiss mice (0.2 mg/kg TETS). The doses were chosen to produce SE with similar time to onset, duration and severity in the two strains. Since the SE was similar, the more persistent damage observed in the C57BL/6J mouse may be due to dose-dependent toxicodynamic effects of TETS. Distinguishing the relative contributions of dose *vs*. strain to differences in neuropathology observed in NIH Swiss mice *vs*. C57BL/6J mice will require further study.

In conclusion, TETS-induced SE of ~40 min in duration caused a delayed and transient neuropathological response, the spatiotemporal profile and magnitude of which varied between NIH Swiss and C57BL/6J mice. Collectively, these data suggest that the extent and persistence of neuropathologic changes following TETS-induced SE is likely influenced by genetic factors and/or the magnitude of exposure. One unique animal that experienced TETS-induced SE of ~120 min exhibited much more severe neuropathology, suggesting that seizure duration may also influence neuropathological consequences. Our observations suggest that the therapeutic window for effectively mitigating the neurological consequences of acute TETS intoxication using antiseizure medication may be longer than for other chemical convulsants, such as OPs. However, TETS-intoxicated patients who either do not respond to standard of care medical countermeasures or receive such medications at significantly delayed times post-exposure, are at increased risk for more extensive and severe neuropathology.

## Supplementary Material

Supplemental material

## Abbreviations:

ADC: apparent diffusion coefficient
DWI: diffusion-weighted imaging
MDZ: midazolam
MRI: magnetic resonance imaging
OPs: organophosphates
PAM: positive allosteric modulator
PET: positron emission tomography
SE: *status epilepticus*
SUV: standardized uptake value
T2W: T_2_-weighted
TETS: tetramethylenedisulfotetramine
TSPO: translocator protein
VEH: vehicle
