# Supplementary material for: Strain differences in the extent of brain injury in mice after tetramethylenedisulfotetramine-induced status epilepticus
Source: Neurotoxicology. Author manuscript; Available in PMC 2021 Dec 1. (PMC8595842; doi:10.1016/j.neuro.2021.08.011)
Supplement: Supplemental material [file NIHMS1745160-supplement-Supplemental_material.docx]

**Supplemental Material**

**Strain and seizure duration influence the extent of brain injury in mice after tetramethylenedisulfotetramine-induced *status epilepticus***

Jonas J. Calsbeek^1^*, Eduardo A. González^1^*, Casey A. Boosalis^1^, Dorota Zolkowska^2^, Donald A. Bruun^1^, Douglas J. Rowland^3^, Naomi H. Saito**^4^**, Danielle J. Harvey**^4^**, Abhijit J. Chaudhari^3^, Michael A. Rogawski^2^, Joel R. Garbow^5^, Pamela J. Lein^1^

^1^Department of Molecular Biosciences, University of California, Davis, School of Veterinary Medicine, Davis, CA 95616, USA ([jcalsbeek@ucdavis.edu](mailto:jcalsbeek@ucdavis.edu), [azgonzalez@ucdavis.edu](mailto:azgonzalez@ucdavis.edu), [caboosalis@ucdavis.edu](mailto:caboosalis@ucdavis.edu), [dabruun@ucdavis.edu](mailto:dabruun@ucdavis.edu), [pjlein@ucdavis.edu](mailto:pjlein@ucdavis.edu)); ^2^Department of Neurology, University of California, Davis, School of Medicine, Davis, CA 95616, USA ([dzolkowska@ucdavis.edu](mailto:dzolkowska@ucdavis.edu), [rogawski@ucdavis.edu](mailto:rogawski@ucdavis.edu)); ^3^Center for Molecular and Genomic Imaging, University of California, Davis, College of Engineering, Davis, CA 95616, USA ([djrowland@ucdavis.edu](mailto:djrowland@ucdavis.edu); ajchaudhari@ucdavis.edu); ^4^Department of Public Health Sciences, University of California, Davis, School of Medicine, Davis, CA 95616, USA ([djharvey@ucdavis.edu](mailto:djharvey@ucdavis.edu), [nhsaito@ucdavis.edu](mailto:nhsaito@ucdavis.edu)); ^5^Biomedical Magnetic Resonance Laboratory, Mallinckrodt Institute of Radiology, Washington University in St. Louis, School of Medicine, St. Louis, MO 63110, USA ([garbow@wustl.edu](mailto:garbow@wustl.edu)).

*These authors contributed equally to this manuscript.

Corresponding Author:

Dr. Pamela J. Lein

Department of Molecular Biosciences

University of California Davis School of Veterinary Medicine

1089 Veterinary Medicine Drive, 2009 VM3B

Davis, CA 95616

Telephone: (530) 752-1970 Fax: (530) 752-7690

Email: [pjlein@ucdavis.edu](mailto:pjlein@ucdavis.edu)

**Table S1.** Mean Apparent Diffusion Coefficient by strain, exposure group, timepoint, and brain region.

**Table S2.** Mean standard deviation in ADC by strain, exposure group, timepoint, and brain region. ****

**Table S3:** Average standardized uptake values by strain, exposure group, timepoint, and brain region.
